# Examining the quality of news media reporting of complex mental illness in relation to violent crime in Australia

**DOI:** 10.1177/00207640231194481

**Published:** 2023-08-29

**Authors:** Madeline Graham, Amy Morgan, Elizabeth Paton, Anna Ross

**Affiliations:** 1 Melbourne School of Population and Global Health, University of Melbourne, VIC, Australia; 2 Everymind, Newcastle, NSW, Australia; 3 School of Humanities, Creative Industries and Social Sciences, University of Newcastle, NSW, Australia

**Keywords:** Complex mental illness, news media, media reporting, media coverage, stigma, psychosis, crime

## Abstract

**Purpose:**

News reporting on mental illness can perpetuate stigma. An understanding of the current picture of such reporting is important to identify areas for improvement. This study investigated the quality of Australian news media coverage of complex mental illness in the context of crime and violence over a 2-year period, prior to the release of new media guidelines.

**Methods:**

This research utilised a systematic search of Australian news articles that were published between July 2018 and July 2020 and reported on mental illness in relation to violent crime. Researchers developed a Mental Illness and Crime Reporting Quality Framework to determine quality scores for news articles according to 11 relevant factors in media guidelines. An additional 11 characteristics of articles were extracted for further descriptive analysis.

**Results:**

One-hundred and twenty-eight Australian news articles met inclusion criteria. The average quality score was 50 (*SD* = 13.91) out of a possible maximum score of 100 (range 11–78). Strengths and weaknesses were identified as some criteria were consistently met, and other criteria were met rarely or not at all. There were emerging trends between quality scores and article characteristics, including publication source, though these analyses were not statistically significant.

**Conclusion:**

The findings indicate that Australian news coverage of complex mental illness and violent crime met half of the criteria of reporting guidelines that minimises risk of perpetuating or reinforcing stigma. This demonstrates significant opportunity to improve the overall quality of media reporting on crime and mental illness. Future research should evaluate the impact of the guidelines on the quality of news reporting after their implementation by utilising a similar methodology, using these findings as a baseline measure.

## Introduction

### Stigma associated with complex mental illness

Complex mental illness includes illnesses where symptoms of psychosis may be experienced, including schizophrenia and bipolar disorder (Morgan et al., 2014). In addition to the inherent challenges that may come with managing an illness, people with complex mental illness also experience stigma and discrimination. Stigma can be defined as an attribute and associated stereotype that is seen as negative amongst the society one is in (Goffman, 2009). Stigma can lead to discrimination, lack of support and empathy, and can reduce help-seeking for symptoms and treatment engagement, which may impact an individual’s ability to find work and be socially included (Rüsch et al., 2005). The BETA National Survey of Mental Health-Related Stigma and Discrimination found those who experience more complex mental health issues are more likely to experience stigma and discrimination, with nine out of ten survey respondents reporting any kind of discrimination (Behavioural Economics Team of the Australian Government [BETA], 2022). People with complex mental illness may also internalise that stigma, impacting their self-image and view of their own capabilities, including management of their illness and perceived potential for recovery (Rüsch et al., 2005). This can negatively impact self-esteem and contribute to other psychological challenges such as depression (Boyd et al., 2014).

Specific stereotypes, assumptions and the nature of stigma generally differs between types of mental illness. People with complex mental illness may be considered dangerous, incompetent, unpredictable, or more violent than those without a mental illness (Griffiths et al., 2006; Ross et al., 2019). Nee and Witt (2013) showed that people thought those with mental illness were more likely to commit a crime than those without, with stereotypical reasoning. These beliefs are inconsistent with data, which demonstrates the population-attributable risk factor for people with complex mental illness in perpetrating violence generally falls between 4% and 6% (Fazel & Grann, 2006; Walsh et al., 2018). While symptoms of complex mental illness can precipitate violence, they are not a predictor in their own right (Appelbaum et al. (2000). People living with complex mental illness are far more likely to be victims than perpetrators of violence (Stuart, 2003). While there is a small increased risk of violent behaviour associated with untreated psychosis (Douglas et al., 2009; Witt et al., 2013), this elevated risk is significantly smaller than the risk associated with being male, single, having low socioeconomic status or using substances (Hiday, 1995; Hinshaw & Stier, 2008). Social factors of poverty and breakdown of family institutions, combined with antisocial personality traits, substance use and an environment of violence, are the most common causes of violence, usually occurring in tense and stressful circumstances (Hiday, 1995; Stuart, 2003; Vogel, 2014).

### Media’s role in stigma

As both complex mental illness and occurrences of violence are rare, which limits first hand exposure, the association between violence/dangerousness and complex mental illness is informed by media depictions (Reavley et al., 2016; Stuart, 2003). Previous research has detailed the impact of media portrayals of complex mental illness on stigma. Ross et al. (2019) found that positive news portrayals of mental illness can reduce stigmatising attitudes, whereas negative portrayals, including inaccurate stereotypes such as violence, sensationalising situations, or using demeaning language, can increase stigma. Both fictional and news media can reinforce the association between mental illness and violence (Parrott & Parrott, 2015). This is often done by emphasising potential danger, and rarely portraying people with mental illness as the victim, despite this being more common (Hinshaw & Stier, 2008).

Additionally, people with mental illness are rarely framed in a positive light in news reporting discussing mental illness, which means the public’s exposure to the reality of lived experience through the media is limited (Klin & Lemish, 2008; Nairn & Coverdale, 2005). Cain et al. (2014) found that in print and online media reports, schizophrenia was heavily associated with crime, contributing to social misunderstanding of complex mental illness, increasing stigma. As such, the improvement of the quality of media reporting is essential to help reduce stigma (Hocking, 2003).

### Initiatives to reduce stigma in media portrayals of mental illness in Australia

Everymind’s Mindframe programme, funded through the Australian Government Department of Health, aims to promote accurate and balanced representation of mental illness and suicide (Everymind, 2022). The Mindframe guidelines for responsible reporting of mental health topics provide guidance and training to journalists and other media professionals to increase their understanding of potential issues related to reporting on mental illness and suicide, and increase their knowledge and confidence in reporting responsibly to reduce stigma (Skehan et al., 2012). The guidelines and training on responsible reporting on suicide and mental illness were released in 1999 and redeveloped by Everymind in 2002, and have been found to be an effective intervention to improve the quality of media portrayals of mental illness and suicide (Pirkis et al., 2009). Other jurisdictions have similar reporting guidelines, including Canada’s Mindset (2020), UK’s *Time to Change* (2020) guidelines, Ireland’s *Headline* (2020) guidelines, Mental Health Foundation of New Zealand’s (2018) media guidelines and Singapore National Council of Social Services (2021). However, the Mindframe programme is considered to be world-leading in its approach to media advocacy, active dissemination and collaborative approach to working with media professionals to improve news reporting on suicide (Bohanna & Wang, 2012).

Despite this emerging evidence regarding the effectiveness of media guidelines on improving the quality of media coverage of more prevalent mental illness such as depression and anxiety, media portrayals of complex mental illness are yet to experience similar improvements, both in Australia and internationally (Anderson et al., 2020; Hildersley et al., 2020; Pirkis et al., 2009). Aiming to improve the quality of these portrayals, Mindframe’s *Guidelines on media reporting of severe mental illness in the context of violence and crime* were released in 2020 (Everymind, 2020; Ross et al., 2020). These were developed collaboratively using the Delphi expert consensus methodology, with media and mental health professionals, as well as people with lived experience of complex mental illness, forming the expert panels to determine their content. These best practice guidelines advise media on responsible and balanced reporting of complex mental illness in the context of crime, aiming to limit stigmatising elements in news reports and ultimately reduce discrimination (Everymind, 2020). These guidelines are unique in their specificity to violence and crime, being the first of their kind to be developed globally.

In order to evaluate any improvements in the quality of news reporting generated by these newly released media guidelines, it is important to first understand the current quality of such portrayals. Therefore, this study aims to determine the quality of Australian news portrayals of complex mental illness and crime by systematically comparing reporting against the best practice guidelines, in the 2 year period before their release.

## Materials and methods

### Study design

This study was a baseline content analysis of Australian news articles resulting from a systematic search of a news database.

### Systematic search

The ProQuest database ANZ Newsstream was searched for news articles published between 1 July 2018 and 1 July 2020. This time period was chosen in order to obtain and assess the quality of news articles reporting on complex mental illness in the context of crime before the Mindframe guidelines were released in August 2020. The ANZ Newsstream database includes articles that appeared in print and online formats, from a variety of Australian news sources. The sample was limited to news articles reporting on complex mental illness specifically and included violent crime events.

A systematic search strategy was developed based on a sample of 17 news articles that reported on mental illness in the context of crime. These were further refined following 18 test searches in the database to return the most relevant results. The final search terms were: mental health; mental illness*; mental disorder*; bipolar; psychosis; psychotic; schizophrenia; schizophrenic; paranoid; paranoia; crim*; violen*; assault*; kill*; murder*; police; court; excluding the term ‘legalising’ and letters to the editor. Additional filters were applied to limit the results to Australian news articles only and news sources (excluding correspondence and general information).

The inclusion criteria were: article must mention violent crime involving victim(s) AND complex mental health issues (mental illness, mental disorder, bipolar disorder, schizophrenia, psychosis, paranoia, borderline personality disorder); the article must report on an event OR report on the relationship between crime or violence and mental illness; the article contains speculation or proof of mental impairment related to crime; mentions perpetrator mental health issues (not drug-induced psychosis); article is from a major or reputable news source (no independent blogs); article is unique (non-duplicate across syndicated news sources); is available online; is a full text article; published after July 2018 and before July 2020; and from an Australian publication.

The decision to exclude articles that mentioned the perpetrator experiencing drug-induced psychosis was made because content discussing alcohol and other drugs is covered by Mindframe for *Alcohol* and Other Drugs: Guidelines for communicating about alcohol and other drugs, released in 2019. These guidelines aim to reduce stigma associated with alcohol and drug use, and cover how to communicate about someone using drugs, language advice, and specific help-seeking information (Mindframe, 2022). Content that only discussed mental illness (usually psychosis) as a result of drug use and independent from complex mental illness was considered to be outside the scope of the current study as it is not related to the *Guidelines on media reporting of severe mental illness in the context of violence and crime*.

**Figure 1.**
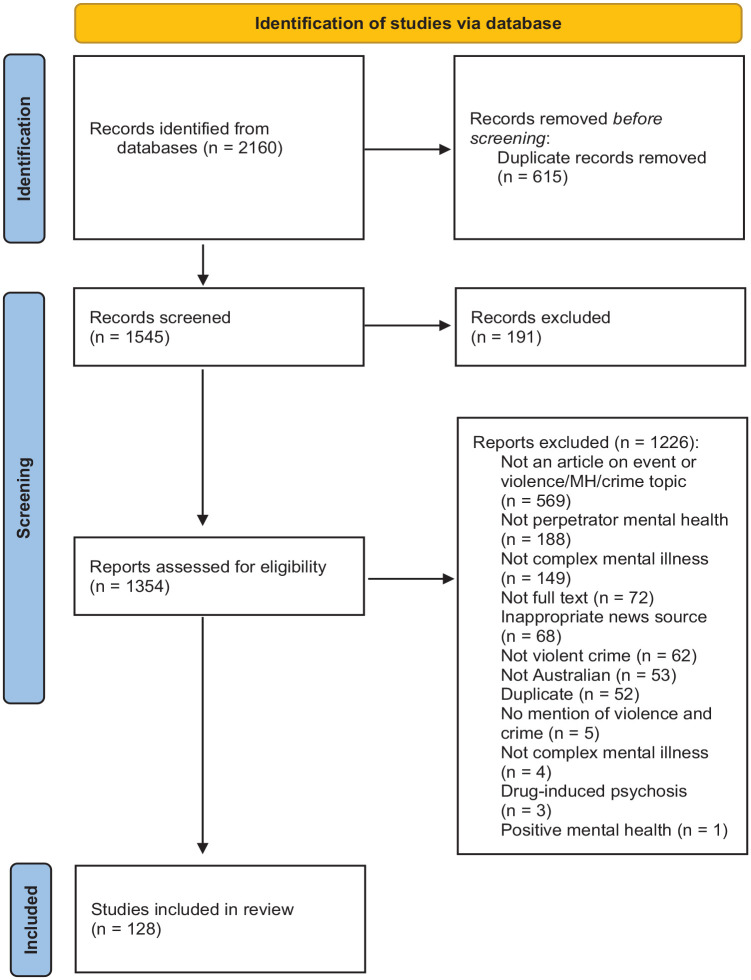
PRISMA flow diagram of the screening process for article inclusion.

This search returned 2,160 results which were screened for eligibility using systematic review software, Covidence (Veritas Health Innovation, 2022). This screening process is shown in Figure 1. Ten percent (*n* = 271) of news articles were also doubled-screened by two researchers (MG, AR) to ensure the study inclusion criteria were being applied consistently.

### Measures

In total, 128 news articles met the inclusion criteria. Articles that met the inclusion criteria were scored against the Mental Illness and Crime Reporting Quality Framework, which was developed by the researchers for use in this study, and is the first of its kind for examining the quality of news media reporting. The Quality Framework is based on key principles of the Mindframe *Guidelines on media reporting of severe mental illness in the context of violence and crime* (Everymind, 2020; Ross et al., 2020). It was adapted for the analysis of text-based media items with no accompanying images or videos, as these were not available through the database used in this study. The descriptions used in the guidelines were also adapted for the purposes of scoring ease, with examples added to the criteria to assist with scoring. This included rephrasing key principles that were phrased negatively to reduce confusion for researchers over double negatives. For example, ‘do not use stigmatising language’ was rephrased as ‘used stigmatising language’ and then reverse scored in determining the overall quality score for a news article. Instructions for the interpretation and application of each criteria were developed to increase consistency in scoring these (Supplemental Appendix A). The coding framework was then tested by five rounds of double coding with two reviewers. Revisions were made to the scoring framework and instructions for use after each round to address inconsistencies in scoring that were occurring between the two researchers. The researchers double-scored 62.5% of articles to improve reliability, with an average inter-rater reliability Kappa score of 0.7 (Supplemental Appendix B). The 11 scored factors and 11 characteristics of articles that comprise the Mental Illness and Crime Reporting Quality Framework are displayed in Table 1 below.

**Table 1.** Overview of items in Mental Illness and Crime Reporting Quality Framework.

Scored factors	
Factor	Description
Used authoritative sources for mental illness status	The article uses court reports, police reports or the statements of mental health professionals to assert whether the perpetrator had a mental illness.
Implied all people with mental illness are violent or a risk to the community*	The article makes broad generalisations about mental illness that do not speak to a specific person.
Provided details of other relevant factors contributing to the incident	Mental illness status is not the only factor mentioned in the article that could be associated with violence. For example, drug use or a history of violence.
Mental illness was inferred or suggested to be the main cause of violence*	Mental illness mentioned in isolation, with no other contextual factors around incident, and the proximity of mental illness and violence or the emphasis on mental illness throughout the article suggest it was the cause.
Provided additional information from health professionals about mental illness	The article includes quotes or information provided by mental health professionals about the nature of mental illness.
Provided descriptions of the consequences for verdicts of ‘not guilty by mental impairment’ or ‘act proven but not criminally responsible’ (NSW only)	Where a verdict of this nature is being reported on, any restrictions or requirements of the defendant/perpetrator are also included, such as detainment in psychiatric hospitals.
Distinguished mental illness claims made by lawyers in the process of defence from official medical diagnoses of mental illness	Where a mental illness claim is made by a defence lawyer, it is made clear that this is a defence/legal claim, not confirmation or equivalent to a diagnosis from a medical professional.
Used person-first language	The article uses appropriate language when discussing a person with mental illness. For example, ‘person with schizophrenia’ as opposed to ‘schizophrenic’.
Used stigmatising language*	The article uses stigmatising terms including colloquialisms, sensationalist language and custodial terms in place of medical terms. For example, ‘maniacal’, ‘crazed’, ‘deranged’.
Provided help seeking information	The article links to appropriate help seeking resources such as SANE or Lifeline.
Provided link to further information about mental illness and crime	The article links to authoritative literature, articles and resources that explain the link between mental illness and crime in more detail.
*Characteristics collected*	
Publication source	The publisher who owns the paper.
Audience	A metro (major city) or regionally circulated paper (all other areas).
First mention of mental illness	Where the first mention of mental illness occurred, either in the headline, first paragraph or body of the article.
Type of mental illness	If the article specifies the complex mental illness (schizophrenia, psychosis, bipolar disorder) or not (unspecified).
Perpetrator gender	What is the gender of the perpetrator (male/female)?
Treatment status	Is any previous or current treatment mentioned e.g. ‘the perpetrator was discharged from a psychiatric hospital last month’?
Story type	Is the story reporting on a current event (ongoing report), singular coverage of an event or stand-alone piece on the topic of mental illness and crime (one-off), or a historical event (not currently before courts)?
Non-authoritative source of mental illness status	What was the source of the perpetrator’s mental illness status, if it was not from an authoritative source e.g. family members?
Other factors mentioned	Where other relevant factors are included that describe the perpetrator’s social context, such as drug use or refugee status.
Stigmatising language quoted	Where stigmatising language was used, was it said by someone else and quoted in the article or used by the journalist?
Event name	The perpetrator’s name, or the name of the event (e.g. Gargasoulas, Bourke Street attack) to identify recurring coverage of such incidents.

* Criteria that were negatively scored.

### Statistical analysis

Scored factors could be marked as yes, no, can’t tell, or not applicable (NA). An article’s possible quality score was calculated based on the total number of items scored, excluding those that were marked ‘not applicable’ or ‘can’t tell’ during the scoring process. Eight factors contributed positively to the quality score (counted if ‘yes’), and the remaining three were reverse scored due to negative phrasing in the scoring framework (counted if ‘no’). These scores were added together then divided by the total possible score to give the final quality score, which was then converted into a score out of 100.

Data was analysed in IBM SPSS Statistics 27 (IBM Corp, 2020) to determine frequencies of quality scores, scored factors, and descriptive characteristics. Analysis of variance was used to explore whether quality scores varied according to where the first mention of mental illness was, the article type, publication source and type of mental illness in the article. It was hypothesised earlier mentions of mental illness in articles would result in lower quality scores, with sensationalised headlines being used for clickbait. Article type and type of mental illness were analysed to identify specific opportunities for training and improvement, and specific stereotypes associated with different types of mental illness, respectively. Reporting quality and publication source was analysed to determine differences in average reporting quality between different news outlets. Two of the major Australian publishers, NewsCorp and Nine Entertainment Co.,^1^ both publish broadsheet and tabloid publications. Australian Associated Press (AAP) is a newswire service and online publisher, and the Australian Broadcasting Corporation (ABC) publishes online news on their own platforms.

## Results

### News article characteristics

The vast majority of the news articles featured a male perpetrator (85.2%). One in five (19.5%) news articles in the sample reported on the Melbourne Bourke Street incident perpetrated by James Gargasoulas. This occurred in January of 2017 where the perpetrator drove a car into pedestrians along Bourke Street (a busy pedestrian mall) in the city of Melbourne, resulting in six deaths and 27 seriously injured. Subsequent reporting on this event and the criminal trial in 2018 frequently mentioned the perpetrator’s previous diagnosis of schizophrenia.

### Quality scores for news articles

The mean and median quality scores of news articles reporting on complex mental illness and violence were both 50 (*SD* = 13.91) out of a maximum score of 100. The lowest quality score was 11 and the highest score achieved was 78, reflecting a varied range of quality factors met by the news articles.

An overview of scoring on each quality criteria is outlined below in Table 2. The sample of news articles tended to report well on several criteria, including the use of authoritative sources for mental illness status, providing details of other relevant factors, and the use of person-first language. This occurred in 80%, 70% and 75% of articles respectively, where relevant. Additionally, implying all people with mental illness are violent or a risk to the community, and using stigmatising language, were commonly avoided. Only 10% of articles implied all people with mental illness are violent or a risk to the community, and 25% used stigmatising language.

**Table 2.** Overview of scoring for included news items (*N* = 128).

Score	Authoritative source	Implies all people are violent*	Details of relevant factors	Inferred main cause of violence*	Additional information from health professional	Descriptions of verdicts	Distinguishes lawyer claims	Person-first language	Stigmatising language*	Help seeking information	Further information on mental illness and crime
Yes	102 (79.68%)	13 (10.16%)	90 (70.31%)	85 (66.41%)	6 (4.69%)	10 (7.81%)	18 (14.06%)	96 (75%)	33 (25.78%)	16 (12.5%)	0 (0%)
No	22 (17.19%)	115 (89.84%)	37 (28.91%)	43 (33.59%)	121 (94.53%)	5 (3.91%)	0 (0%)	20 (15.63%)	95 (74.22%)	112 (87.5%)	128 (100%)
N/A	3 (2.34%)	0 (0%)	1 (0.78%)	0 (0%)	1 (0.78%)	113 (88.28%)	109 (85.16%)	12 (9.38%)	0 (0%)	0 (0%)	0 (0%)
Can’t tell	1 (0.78%)	0 (0%)	0 (0%)	0 (0%)	0 (0%)	0 (0%)	1 (0.78%)	0 (0%)	0 (0%)	0 (0%)	0 (0%)

* Criteria that were negatively scored.

Criteria that could contribute to a higher quality score that were most often overlooked in news reports were providing information from health professionals about mental illness, providing help-seeking information, and providing links to further information about mental illness and violence, which were included in only in 5%, 13% and 0% of articles respectively. Implying or suggesting mental illness as the main cause of violence was the biggest negative influence on quality scores, occurring in 66% of articles.

Providing descriptions of the consequences for verdicts of ‘not guilty due to mental impairment’, and distinguishing mental illness claims made by lawyers, were only relevant in a few instances of court reporting and therefore did not greatly influence quality scores overall. These factors were not applicable in 88% and 85% of articles respectively.

### Associations between quality scores and article characteristics

The first mention of mental illness more frequently occurred in the body of the news article (78.1%), followed by mentions in the first paragraph (14.8%) and mentions in the headline (7%). Although the mean quality score of articles of headline and first paragraph mentions was lower (quality score of 44) than body text mentions (quality score of 56), this difference was not found to be significant (*F*(2, 125) = 2.86, *p* = .061).

Additionally, differences in quality scores between types of news items was explored. Reports on ongoing events were the most common type, followed by one-off coverage of incidents or stand-alone pieces on the topic, and reports on historical incidents (75.8%, 20.3% and 3.9% respectively). Ongoing reports were generally of higher quality with a median score of 56 out of 100, followed by one-off articles with a median score of 50, with historical articles having the lowest median score of 44. These differences were not found to be statistically significant (*F*(2,125) = 1.76, *p* = .177).

Quality scores according to news publisher are presented in Table 3. The majority of articles in the sample were from three publishers: AAP (39.1% of articles); NewsCorp (38.3%); and Nine Entertainment Co. (18.8%), with ABC and Australian Community Media only contributing to 3.1% and 0.8% (or one article in the sample) of articles respectively. Mean quality scores were fairly similar across these publishers, (*F*(2,125) = 1.51, *p* = .226), excluding ABC and Australian Community Media due to low frequency.

**Table 3.** Average quality scores of news items and frequency of stigmatising language by publisher.

	AAP	NewsCorp	Nine Entertainment Co	ABC	Australian Community Media
Mean quality score (out of 100)	52.33	47.84	51.25	50.00	70.00
Std. deviation	11.86	16.44	12.66	6.41	N/A
Frequency of stigmatising language (yes *n*, %)	9 (26%)	17 (50%)	6 (18%)	1 (3%)	1 (3%)
Frequency of stigmatising language (no *n*, %)	41 (44%)	32 (34%)	18 (19%)	3 (3%)	0
Frequency of stigmatising language (total)	50	49	24	4	1

Table 3 also shows the use of stigmatising language by publisher. Sources differed in their use of stigmatising language, with NewsCorp articles having the highest proportion of use, contributing to over 50% of the total use of stigmatising language. There was no significant difference in mean quality score (inclusive of all measures) across publishers (*F*(2,125) = 1.505, *p* = .226), excluding ABC and Australian Community Media due to low frequency.

Data were also assessed to determine if there were differences in quality scores between the types of mental illnesses reported on, presented in Table 4. Schizophrenia was the most frequently reported complex mental illness in relation to crime, with 58% of articles in the sample mentioning this diagnosis. Unspecified mental illness was the next most frequent, mentioned in 34% of news articles. Psychosis, bipolar disorder or borderline personality disorder only occurred in 5.5%, 1.6% and 0.8% of articles respectively. Quality scores across the five different mental illness types did not significantly differ, (*F*(4,123) = 2.34, *p* = .590).

**Table 4.** Average quality scores of news items by type of mental illness (*N* = 128).

	Schizophrenia	Unspecified	Psychosis	Bipolar	BPD
Frequency of mental illness mention	74 (57.8%)	44 (34.4%)	7 (5.5%)	2 (1.6%)	1 (0.8%)
Mean quality score (out of 100)	52.15	49.39	37.30	50.00	56.00
Std. deviation	12.53	15.25	14.84	7.86	N/A

*Note*. BPD = borderline personality disorder.

## Discussion

This study aimed to determine the quality of Australian news reporting on mental illness and violent crime over a 2-year period from 2018 to 2020. Relevant news articles were scored against the Mental Illness and Crime Reporting Quality Framework and strengths and weaknesses in reporting were identified. Overall, there was large variation in the quality of reporting, but on average articles met only half of the quality criteria outlined in the Mindframe *Guidelines on media reporting of severe mental illness in the context of violence and crime* (Everymind, 2020). These findings indicate that there is significant room for improvement in the quality of reporting of complex mental illness and violence. Strengths in reporting were using authoritative sources for mental illness status, providing details of other relevant contextual factors, and using person-first language.

These findings are novel but not surprising. This study confirms the findings of Cain et al. (2014) and Klin and Lemish (2008) that people with complex mental illness are represented in news coverage as dangerous or violent, as evidenced by the emphasis of this fact in some articles reporting on violent crime. Improving the language and providing more context around discussions of incidents of crime that involve mental illness are important in improving the quality of news coverage in this context. Suggesting that mental illness is the main cause of violence presents the biggest opportunity for potential improvement, due to the frequency of this occurring (66% of articles) and the known negative impacts this can have on public attitudes towards people with complex mental illness (Ross et al., 2019). This perpetuates the idea that mental illness may be a primary driver of violence, despite evidence suggesting other sociodemographic factors, such as being male and having a low socioeconomic status, and substance use are stronger predictors of violent behaviour (Hiday, 1995; Hinshaw & Stier, 2008). Using stigmatising language (such as ‘crazed’, ‘deranged’, ‘released’ from treatment) and implying all people with mental illness are violent should also be avoided to contribute to higher quality reporting. Additionally, the absence of help-seeking information is consistently identified as a key area for improvement to media portrayals of mental illness and suicide more broadly (Francis et al., 2004; Pirkis et al., 2009; Ross et al., 2022).

The distribution of the quality scores in this study demonstrates the variance in the quality of news coverage of mental illness and violent crime. The large spread of scores could suggest that journalists and other media professionals have a diverse range of experiences and understandings of complex mental illness and its relationship to violence and crime. Reductions in the numbers of journalists may also lead to fewer who are able to specialise in court or health reporting. It is also important to recognise that journalists are not immune to societal attitudes that are stigmatising towards mental illness. These views may be held by journalists, as a form of unintentional structural stigma, and journalists may rely on the commonality of these attitudes to inform the readers’ perspective and add context to the story, ultimately implying mental illness is the main cause of violence (Maiorano et al., 2017). Both the unfamiliarity of best practice reporting on complex mental illness and the potential for existing stigmatising beliefs to be present in institutions and individuals highlight the need for widespread implementation of these guidelines, along with ongoing training and monitoring, to encourage responsible and balanced reporting of this issue. Training media professionals has previously been shown to be successful at reducing stigmatising attitudes related to complex mental illness (Ross et al., 2019). Journalists and other media professionals need to be empowered to consistently challenge the status quo of historically portraying complex mental illness in a way that suggests that it is inherently related to violence. This includes reducing suggestions in news articles that mental illness is the main cause of violence or that all people with mental illness are violent. These findings do not suggest that incidents of violent crime that involve a person with mental illness and are of public interest should not be reported. Instead, these findings highlight opportunities for this information to be reported in an appropriately nuanced way, to avoid perpetuating stigma associated with complex mental illness.

### Strengths and limitations

While other studies have more broadly examined how complex mental illness is portrayed by Australian media (Cain et al., 2014; Francis et al., 2004; Pirkis et al., 2002), this study is the first to provide a specific understanding of quality of news portrayals that link complex mental illness to violence. This study demonstrates that a scoring framework based on the Mindframe media reporting guidelines (Everymind, 2020) can be used to provide clear insight into the nature of stigmatising content that is commonly found in Australian news coverage. The methodology was rigorous and highly replicable, with the coding framework based on the Mindframe media reporting guidelines which were developed using scientifically-sound expert consensus processes. Additionally, to mitigate the impact of the concentration of the Australian media landscape, which may limit the diversity of ideas presented in reporting on complex and stigmatised topics, such as mental illness (Noam & The International Media Concentration Collaboration, 2016), news coverage was examined from a 2-year time period to ensure a representative sample.

There were also some limitations to this study. The main limitation is that only news content accessible in the ANZ Newsstream database was evaluated. This excludes television and radio coverage of mental illness and violent crime, meaning the impact of audio and visual aids to stories could not be assessed. This database is not exhaustive of all news accessible to and consumed by Australian audiences. Large publishers such as The Guardian and The Daily Mail were not included in this dataset due to their absence from the ANZ Newsstream database, which may have altered the findings. Additionally, the search process could be amended in future studies to add further rigour and ensure all articles were captured by the search strategy, such as, looking at ‘suggested’ or ‘related’ articles in database sidebars. Similarly, all news stories that were evaluated were written in English, and there may be differences in reporting specific to culturally and linguistically diverse communities in Australia. Finally, given the subjective nature of scoring news articles, there is a small risk of bias in interpretation and application of the scoring criteria. However, the majority of articles were double coded and detailed scoring instructions were developed to mitigate this risk.

### Future research

This study expands the existing evidence base by determining the quality of media reporting of complex mental illness in relation to violence and crime, unique to the Australian context. The findings from this study can inform a longitudinal evaluation that measures changes to the quality of news portrayals following the implementation of the *Guidelines on media reporting of severe mental illness in the context of violence and crime* (Everymind, 2020). This study will also inform future evaluations of the effectiveness of media reporting guidelines on mental illness, when applied to complex mental illness, and highlight opportunities for media training. Future research could investigate the principles outlined in the media reporting guidelines that were outside of scope of this research, such as image use and visual representation of these stories. The impact each scored factor has on stigma and how it may contribute to public attitudes should be considered in future research. For example, inferring mental illness is the main cause of violence may reinforce associations between mental illness and violent crime more than the absence of person-first language. Finally, many people obtain their news from a variety of sources, both other news types (radio, broadcast) and international sources, including via social media platforms. Future research should look to these outlets to determine differences in quality of media reporting across channels, and the quality of international news stories and their impacts on Australian audiences.

## Conclusion

The quality of Australian news media reporting on complex mental illness in the context of crime has substantial room for improvement. According to this study based on the Mindframe *Guidelines for news media reporting on complex mental illness in the context of crime* (Everymind, 2020), on average, Australian media articles only met half of the recommended quality criteria to avoid perpetuating stigma. Specific areas for improvement include the provision of information about mental illness, avoiding the use of stigmatising language, and avoiding assumptions about the role of mental illness in articles reporting on complex mental illness and crime. It is recommended that these guidelines be distributed to media outlets, with supporting resources to aid in their adoption and increase general understanding of their importance. Future research should be undertaken to evaluate the impact of the newly developed Mindframe *Guidelines for news media reporting on complex mental illness in the context of crime* (Everymind, 2020) on the quality of news reporting in this context.

## Supplemental Material

sj-docx-2-isp-10.1177_00207640231194481 – Supplemental material for Examining the quality of news media reporting of complex mental illness in relation to violent crime in Australia Click here for additional data file. Supplemental material, sj-docx-2-isp-10.1177_00207640231194481 for Examining the quality of news media reporting of complex mental illness in relation to violent crime in Australia by Madeline Graham, Amy Morgan, Elizabeth Paton and Anna Ross in International Journal of Social Psychiatry

sj-xlsx-1-isp-10.1177_00207640231194481 – Supplemental material for Examining the quality of news media reporting of complex mental illness in relation to violent crime in Australia Click here for additional data file. sj-xlsx-1-isp-10.1177_00207640231194481 for Examining the quality of news media reporting of complex mental illness in relation to violent crime in Australia by Madeline Graham, Amy Morgan, Elizabeth Paton and Anna Ross in International Journal of Social Psychiatry This article is distributed under the terms of the Creative Commons Attribution 4.0 License ( http://www.creativecommons.org/licenses/by/4.0/) which permits any use, reproduction and distribution of the work without further permission provided the original work is attributed as specified on the SAGE and Open Access pages ( https://us.sagepub.com/en-us/nam/open-access-at-sage).

## Notes

1. Nine Entertainment merged with Fairfax Media in December 2018 (Nine Entertainment Co, 2018); in April 2019 they sold off Australian Community Media (Nine Entertainment Co, 2019). This occurred during the analysis period. Regional subsidiaries that form ACM may have carried stories from larger metro papers prior to their being sold off.
