# Supplementary material for: Examining the quality of news media reporting of complex mental illness in relation to violent crime in Australia
Source: Int J Soc Psychiatry. 2023 Aug 29;69(8):2110–20. doi: 10.1177/00207640231194481 (PMC10685681; doi:10.1177/00207640231194481)
Supplement: sj-docx-2-isp-10.1177_00207640231194481 – Supplemental material for Examining the quality of news media reporting of complex mental illness in relation to violent crime in Australia [file sj-docx-2-isp-10.1177_00207640231194481.docx]

## Appendix A - Mental Illness and Crime Reporting Quality Framework

| **Factor** | **Instructions** | **Type** |
| --- | --- | --- |
| Publication source | Who is the owner of this article i.e. NewsCorp, Nine Entertainment - most are listed at the bottom of the article, alternatively look up who owns the paper online. | Characteristic |
| Audience | Major cities are metro, remaining are regional | Characteristic |
| First mention of mental illness | Mental illness, as either the concept or a specific diagnosis i.e. "mental illness" or "schizophrenia" or "unmedicated" | Characteristic |
| Type of mental illness | Select type of mental illness if stated. If it is not stated explicitly i.e. delusions, select unspecified UNLESS description is known to be associated with bipolar disorder, schizophrenia or psychosis exclusively | Characteristic |
| Perpetrator gender | Male or female if gender is revealed in article, unspecified if gender neutral terms are used | Characteristic |
| Treatment | Prior treatment = perpetrator had received treatment at some stage in their life prior to the incident, but were not receiving it at time of incident  Current treatment = perpetrator was receiving treatment at the time of the incident (NOT at time of reporting i.e. after arrest) No treatment described = there is no prior or current treatment described Unable to access treatment = article explicitly states that perpetrator had attempted to access treatment but was not able to | Characteristic |
| Story type | Breaking news = first report of the incident  Ongoing report = following proceedings of the incident i.e. court report  Historical event = referring to an incident that has been finalised or is no longer active i.e. court proceedings delayed indefinitely, or sentence handed down One-off = article is an exposition of an idea/opinion that is not part of normal reporting on an incident | Characteristic |
| Used authoritative sources for mental illness status | Yes = an authoritative source was used, or would have had to have been used based on where the claim came from i.e. the court heard, was receiving treatment, records of, police found, if mentioned in the context of a court proceeding, can be assumed that official records were obtained No = non-authoritative source was used i.e. family or friends or lawyers in the process of defence Can't tell = unclear  N/A = the story does not refer to the mental illness status of a person/persons | Scored |
| Non-authoritative source of mental illness status | Self-reported = if personal expressions of mental illness are used as only source i.e. Facebook status, diaries Family reported = if family expressions of 'they had a mental illness' are used as the only source Authoritative only | Characteristic |
| Implies all people with mental illness are violent or a risk to the community | Separate from main cause of incident Broader generalisations/associations with mental illness, that do not speak about a specific person, re dangerousness/violence i.e. “mothers with psychiatric conditions are at increased risk of” Mental illness over emphasized in relation to violence, dangerousness i.e. “schizophrenia drove them to” | Scored – negative |
| Provided details of other relevant factors contributing to the incident | Mental illness status is not the only factor mentioned in the article that could be associated with violence, i.e. e.g. treatment challenges, substance misuse, history of violence, attitudes towards women, etc | Scored |
| Other factors mentioned | Drugs = mentions of drug use  Refugee = article mentions refugee status  Add more as mentioned in articles | Characteristic |
| Mental illness is inferred or suggested to be the main cause of violence | Mental illness mentioned in isolation, with no other contextual factors around incident Proximity of MI description to violent behaviour (e.g. mentioned in same sentence, first) Emphasis on MI throughout article as cause of violence (e.g. mental illness described earlier in news article compared to other factors; mental illness more commonly occurs/emphasised in news article compared to other contextual factors) Does not apply when no main cause is discernible i.e. the person was just violent/did something harmful | Scored – negative |
| Provided additional information from health professionals about mental illness | Has quotes or information provided by mental health professionals about the nature of mental illness and violence Does not include psychiatrist quotes from court proceedings that confirm/contradict mental illness status - must elaborated on mental illness in relation to violence | Scored |
| Provided descriptions of the consequences for verdicts of ‘not guilty by mental impairment’ or ‘act proven but not criminally responsible’ (NSW only) | Only applicable where a verdict has been handed down  Yes = verdict is explained i.e. this person will not go to prison but will be in a secure mental health facility No = no description but verdict present N/A = no verdict present  Can't tell | Scored |
| Distinguishes mental illness claims made by lawyers in the process of defence from official medical diagnoses of mental illness | Where a mental illness claim is made by a defence lawyer, it is made clear that this is a defence/legal claim, not confirmation or equivalent to a diagnosis from a medical professional Yes = claim from lawyer, article explains/distinguishes this from a diagnosis No = claim from lawyer is not clearly differentiated from medical diagnosis N/A = there is no mental health claim from lawyer Can't tell | Scored |
| Used person-first language | Yes = person-first language is used No = person-first language is not used N/A = the article does not specifically talk about mental illness status | Scored |
| Uses stigmatising language | Yes = language is stigmatising i.e. colloquialisms, sensationalist terms, confuses strong emotions such as rage with mental illness, uses custodial terms instead of medical, “crazed”, “deranged”, “lunatic”, “unhinged”, “shrinks”, “mental institutions”, “released from treatment” No = purely factual information with no editorialising  N/A = no language used associated with mental illness status | Scored – negative |
| Stigmatising language quoted | Stigmatising language is in a quote or statement attributed to a person talking to the journalist, not the journalists' own words | Characteristic |
| Provided help seeking information | https://www.sane.org/  https://www.lifeline.org.au/ | Scored |
| Provided link to further information about mental illness and crime | Example only https://www.betterhealth.vic.gov.au/health/conditionsandtreatments/mental-illness-and-violence | Scored |
| Event name | Perpetrator last name | Characteristic |
